# Ovarian Reserve in Women of Reproductive Age With Sickle Cell Anaemia: A Scoping Review

**DOI:** 10.1002/jha2.70410

**Published:** 2026-10-01

**Authors:** Kehinde Awodele, Sunday Charles Adeyemo, Adeniyi Olanipekun Fasanu, John Oluwadamilola Ojo, Haruna Oche Ahmadu

**Affiliations:** ^1^ Obstetrics and Gynecology Department Osun State Univeristy Osogbo Nigeria; ^2^ Institut Superieur de Sante Niamey Niger; ^3^ Obstetrics and Gynecology Department Uniosun Teaching Hospital Osogbo Nigeria

**Keywords:** anti‐Müllerian hormone, antral follicle count, fertility preservation, follicle‐stimulating hormone, ovarian reserve, reproductive health, sickle cell anaemia

## Abstract

**Background:**

Sickle cell anaemia (SCA) is an inherited haemoglobinopathy disproportionately common among people of African origin, though it affects multiple global populations. Emerging evidence suggests that chronic haemolysis, inflammation and treatment exposures in SCA may affect ovarian function. This scoping review aimed to map available evidence on ovarian reserve in women of reproductive age with SCA.

**Methods:**

PubMed/MEDLINE, Embase, Scopus, Web of Science and CINAHL were searched from inception to 31 January 2026, using Boolean operators combining terms related to SCA, ovarian reserve, anti‐Müllerian hormone (AMH), antral follicle count (AFC) and follicle‐stimulating hormone (FSH). Studies reporting biochemical or radiographic ovarian reserve in reproductive‐aged women with SCA were included. Due to heterogeneous methodologies, data were extracted via standardised forms and narratively synthesised.

**Results:**

Seven studies met the inclusion criteria, comprising six observational studies and one retrospective multi‐centre fertility preservation cohort (total *N* = 488 women with sickle cell disease, of whom the large majority had HbSS). The majority of comparative studies reported lower AMH levels in women with SCA compared to controls, with reductions ranging from 48% to 79% depending on the population and assay method. Direct pooling was not performed due to assay heterogeneity. Lower AFC was also observed in studies reporting this measure. Studies that compared HbSS with HbAA controls consistently reported lower AMH and AFC, whereas data on HbSC and HbSβ° genotypes were too sparse to permit formal genotype comparison. Age demonstrated a stronger negative correlation with ovarian reserve markers among women with SCA compared to controls. Evidence on FSH was limited and inconsistent. Treatment‐related findings, particularly regarding hydroxyurea exposure, were reported but causality could not be established due to confounding by disease severity.

**Conclusion:**

The available evidence indicates that women of reproductive age with SCA, particularly those with HbSS genotype, may demonstrate lower ovarian reserve indices compared to age‐matched HbAA controls, though the cross‐sectional nature of most studies limits causal inference. These findings highlight the need for early reproductive counselling and further longitudinal research.

**Trial Registration:**

The authors have confirmed clinical trial registration is not needed for this submission.

## 1 Introduction

Sickle cell anaemia (SCA) is a genetic haemoglobinopathy resulting from a point mutation in the *β*‐globin gene, leading to the production of haemoglobin S (HbS) [1]. HbS polymerisation under hypoxic conditions causes haemolysis and vaso‐occlusion, resulting in chronic organ damage [1, 2]. The condition is most prevalent in people of African descent, with more than 300,000 affected births globally each year; however, it also affects populations in the Mediterranean, Middle East and India [3]. Advances in care have substantially improved survival, and many women with SCA now live to reproductive age and desire childbearing [2, 3].

Ovarian reserve refers to the reproductive potential of a woman and is typically measured using biomarkers such as anti‐Müllerian hormone (AMH), antral follicle count (AFC) and follicle‐stimulating hormone (FSH) [4, 5]. Emerging evidence suggests that women with SCA may experience reduced ovarian reserve earlier than the general population [5, 6, 7]. This decline could be precipitated by disease‐related exposures, including chronic hypoxia, oxidative stress and microvascular damage, as well as treatment‐related exposures such as hydroxyurea and transfusion therapy [7, 8, 9]. The chronic haemolysis, vaso‐occlusive events, systemic inflammation and cumulative organ injury associated with SCA may collectively impact reproductive health outcomes [1, 2, 3].

Recent research has reported considerably reduced AMH levels and altered AFC in reproductive‐age women with SCA, raising concerns about premature reproductive ageing and reduced fertility potential [5, 7, 10]. Moreover, fertility preservation has emerged as a growing clinical priority, particularly before disease‐modifying therapies, underscoring the importance of ovarian reserve evaluation [2, 7]. Despite increasing interest, the evidence base remains fragmented, with inconsistencies in study design, outcome measures and population characteristics.

A scoping review is required because the evidence on SCA and ovarian reserve, though growing, remains fragmented across various study designs and outcome measures, limiting the ability to make evidence‐based decisions on fertility counselling and preservation. This review maps the extent, range and nature of available evidence to identify gaps and inform future research.

*Research Question*:

What evidence exists on ovarian reserve in women of reproductive age with SCA?

*Aim*:

To map existing evidence on ovarian reserve in women of reproductive age with SCA.

*Objectives*:

(1) To identify studies assessing ovarian reserve in reproductive‐age women with SCA.
(2) To summarise ovarian reserve markers and related outcomes reported.
(3) To highlight evidence gaps to inform future research and clinical practice.

## 2 Methods

### 2.1 Study Design

This scoping review was conducted to map the scope of available evidence on ovarian reserve in reproductive‐age women with SCA. The review was conducted according to the methodological framework of Arksey and O'Malley, expanded by Levac et al., and reported following the PRISMA Extension for Scoping Reviews (PRISMA‐ScR) guidelines [11, 12, 13].

### 2.2 Terminology

In this review, SCA denotes the homozygous HbSS genotype together with the clinically comparable HbSβ° thalassaemia genotype, in line with standard haematological usage. Sickle cell disease (SCD) is used only as the umbrella term for all clinically significant sickling genotypes, that is HbSS, HbSβ°, HbSC and HbSβ+. The title, research question, aim, eligibility criteria and conclusions of this review concern SCA. Because several primary studies recruited mixed SCD cohorts, such studies were eligible only where SCA‐specific data were reported separately or where participants with HbSS or HbSβ° formed the large majority of the sample. Where a cited study reported on a mixed cohort, the term SCD is retained in describing its findings so that the genotype composition of the underlying evidence remains transparent; HbSS is used whenever a finding is genotype‐specific.

### 2.3 Protocol and Registration

A review protocol was created a priori to enhance transparency and methodological rigour. The protocol was designed in accordance with PRISMA‐ScR guidelines. Although scoping reviews are not typically registered in PROSPERO, the protocol guided all stages of the review process.

### 2.4 Eligibility Criteria

The population‐concept‐context (PCC) framework was used to define eligibility criteria, as recommended for scoping reviews addressing heterogeneous and exploratory evidence [11, 13].

*Population*: Women of reproductive age (approximately 15–49 years) with a confirmed diagnosis of SCA (HbSS or HbSβ°). Studies of broader SCD cohorts, including HbSC, were eligible only where ovarian reserve data for participants with SCA were reported separately or where participants with HbSS or HbSβ° formed the large majority of the sample.

*Concept*: Ovarian reserve determined by biochemical markers AMH, FSH and/or radiographic measurements, such as AFC. Studies reporting fertility preservation outcomes relevant to ovarian function were also included.

*Context*: Research carried out either in a clinical, community or research‐based environment, in any geographical location.

*Study designs*: Observational studies (cross‐sectional, case‐control, cohort), interventional studies and systematic reviews reporting ovarian reserve outcomes in women with SCA. Studies on fertility preservation were included where they reported ovarian reserve‐related outcomes.

*Inclusion criteria*: Studies evaluating ovarian reserve using biochemical markers, radiographic measures or fertility preservation outcomes in reproductive‐age women with SCA. Both primary and secondary study designs were included.

*Exclusion criteria*: Studies not reporting ovarian reserve outcomes, case reports, opinion pieces and studies in languages other than English.

### 2.5 Sources of Information and Search Strategy

The electronic search strategy was developed to identify relevant literature across multiple databases: PubMed/MEDLINE, Embase, Scopus, Web of Science and CINAHL. These databases were selected to provide comprehensive coverage of biomedical, reproductive and nursing literature [11, 12, 13]. The search was conducted from database inception to 31 January 2026.

Search terms included combinations of SCA, SCD, ovarian reserve, AMH, AFC, fertility and related terms. Boolean operators (AND, OR, NOT) were used to combine keywords and MeSH terms. The full search strategy for PubMed/MEDLINE is provided in Table S1. Searches were limited to English‐language publications; grey literature was not included, representing a potential limitation [11, 12, 13]. Reference lists of included articles and relevant reviews were manually screened to identify additional eligible studies [13, 14, 15].

### 2.6 Study Selection

All retrieved records were imported into reference management software, and duplicates were removed. Titles and abstracts were independently screened by two reviewers (K.A. and S.C.A.) against the eligibility criteria. Full‐text articles were then assessed for inclusion by the same reviewers. Discrepancies were resolved through discussion or consultation with a third reviewer (A.O.F.) when consensus could not be reached. The study selection process was documented using a PRISMA‐ScR flow diagram (Figure 1).

**FIGURE 1:**
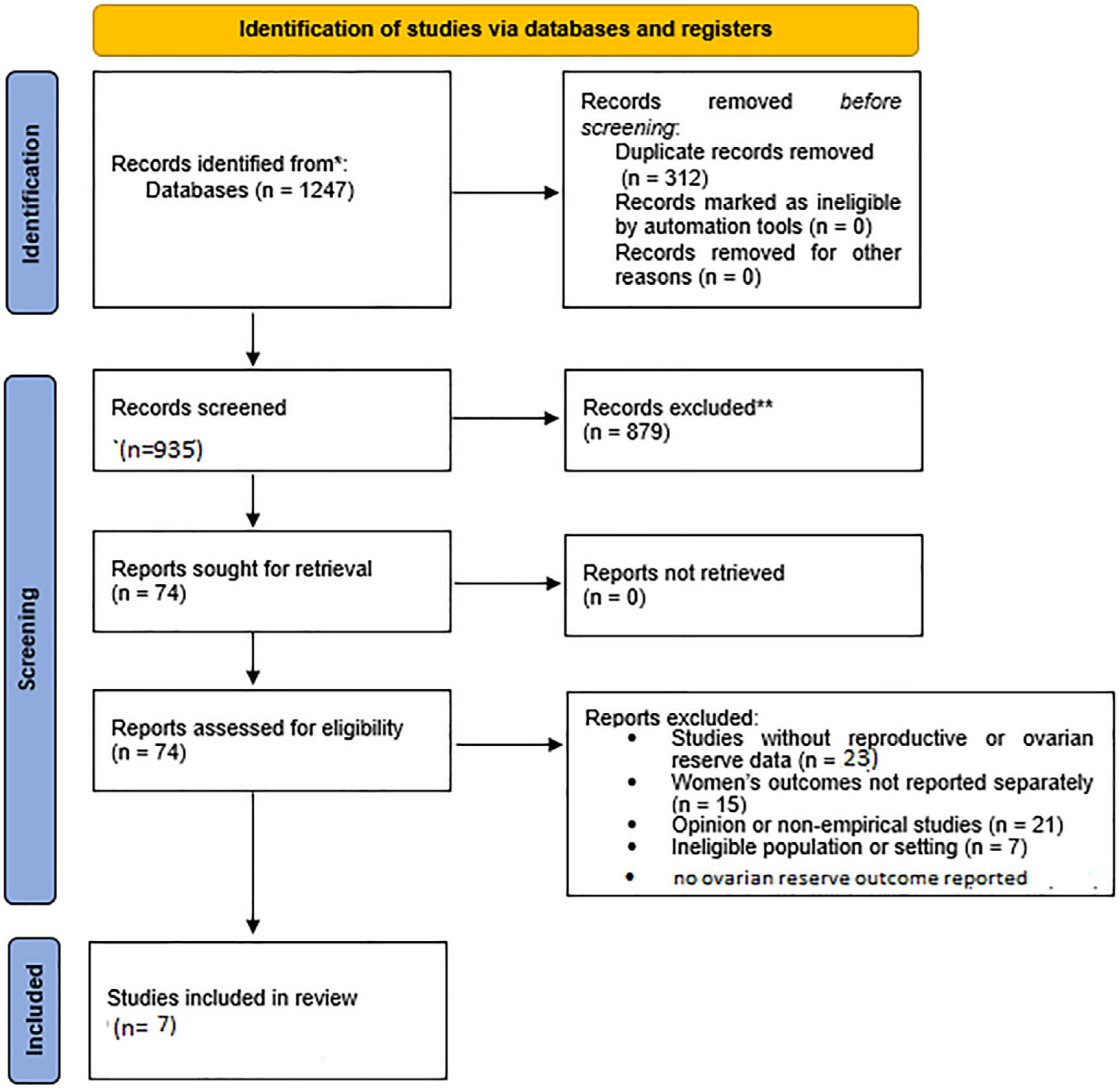
PRISMA‐ScR flow diagram. The figure illustrates the identification, screening, eligibility assessment and inclusion of studies for the scoping review following the Preferred Reporting Items for Systematic Reviews and Meta‐Analyses Extension for Scoping Reviews (PRISMA‐ScR) guidelines. Seven studies were included and charted in Tables 2 and 3.

### 2.7 Data Extraction

A standardised data extraction form was developed and piloted on a subset of included studies. Data extracted were study characteristics (author, year, country and design), participant demographics, SCA genotype, ovarian reserve measures evaluated, treatment exposures (e.g., hydroxyurea, transfusion therapy) and main findings regarding ovarian reserve and fertility outcomes [5, 10].

### 2.8 Data Synthesis

Given the expected heterogeneity in study designs and outcome measures, narrative and descriptive synthesis was employed, consistent with scoping review methodology [11, 12]. Findings were organised thematically based on ovarian reserve parameters and treatment exposures. Subgroup considerations included genotype‐specific results and treatment effects on ovarian reserve where data permitted. Evidence gaps and areas for future research were identified to inform clinical practice and reproductive counselling for women with SCA. The PRISMA‐ScR checklist is presented in Table 1.

**TABLE 1:** PRISMA Extension for Scoping Reviews (PRISMA‐ScR).

**Section**	**Item no**.	**Checklist item**	**Reported on page**
**Title**	1	Identify the report as a scoping review.	1
Abstract	2	Provide a structured summary including background, objectives, eligibility criteria, sources of evidence, charting methods, results and conclusions.	2
Introduction			
Rationale	3	Describe the rationale for the review in the context of existing knowledge.	3
Objectives	4	Provide an explicit statement of the objectives and research questions.	4
Methods			
Protocol and registration	5	Indicate whether a review protocol exists and if it was registered.	5
Eligibility criteria	6	Specify characteristics of sources of evidence (e.g., population, concept, context, study design) and rationale.	5
Information sources	7	Describe all information sources used and date of last search.	6
Search	8	Present the full electronic search strategy for at least one database.	6 (Full strategy in Table S1)
Selection of sources of evidence	9	Describe the process for selecting sources of evidence.	6
Data charting process	10	Describe the methods for charting data from included sources.	7
Data items	11	List and define all variables for which data were sought.	7
Critical appraisal of individual sources	12	If done, provide rationale and describe methods used for appraisal.	Not performed (scoping review)
Synthesis of results	13	Describe methods of handling and summarising the data.	6‐7
Results			
Selection of sources of evidence	14	Provide numbers of sources screened, assessed, included and excluded, with reasons.	7 (Figure 1)
Characteristics of sources of evidence	15	Present characteristics of included studies.	7–8 (Table 2)
Critical appraisal within sources	16	If performed, present appraisal data.	Not performed
Results of individual sources	17	Present relevant data from each included source.	8–10 (Table 3)
Synthesis of results	18	Summarise and present findings across sources of evidence.	10–11
Discussion			
Summary of evidence	19	Summarise main findings and relevance to key groups.	11–12
Limitations	20	Discuss limitations of the scoping review process.	12
Conclusions	21	Provide interpretation of results and future research directions.	12‐13
Funding			
Funding	22	Describe sources of funding and role of funders.	Title page (Page 1)

## 3 Results

### 3.1 Characteristics of Included Studies

A summary of the characteristics of the seven included studies is presented in Table 2, detailing study design, setting, sample size, age range, ovarian reserve markers measured and main outcomes. Publications spanned 2015–2026 and were predominantly observational in nature, including comparative cross‐sectional studies [9, 15], a case‐control study [4], cross‐sectional and cohort studies [5, 8, 10] and a retrospective multi‐centre study of fertility preservation outcomes [7]. A fertility‐knowledge survey of adults with SCD [6] was retrieved by the search but reported no ovarian reserve biomarker; it was therefore not charted as an included study and is referred to only as contextual literature, so that the study count in the text, the PRISMA‐ScR flow diagram (Figure 1) and Tables 2 and 3 all correspond to seven studies.

**TABLE 2:** Characteristics of included studies.

**Author (year)**	**Country**	**Study design**	**Population (*n*)**	**Age range**	**Genotype**	**Ovarian reserve measures**
Kopeika et al. [4]	UK	Case‐control	50 SCD vs. 73 controls	Reproductive age	SCD (not specified)	AMH
Shandley et al. [10]	USA	Cross‐sectional comparative	152 SCD vs. 128 controls	20–45 years	SCD	AMH
Pecker et al. (2022)	USA	Observational cohort	26 women with SCA	19–30 years	HbSS	AMH, FSH, AFC
Goldenberg et al. [7]	USA (multi‐centre)	Retrospective cohort	46 SCA (55 COH cycles)	Median 23.7 years	93% HbSS	AMH, oocyte yield, E2
Adewunmi et al. [15]	Nigeria	Comparative cross‐sectional	75 HbSS vs. 75 HbAA	Reproductive age	HbSS	AMH, AFC, oxidative stress markers (SOD, GPx, MDA)
Elchuri et al. [8]	USA	Cross‐sectional	56 females (14 SC, 33 HU, 9 BMT)	10–21 years	HbSS/Sβ°	AMH, FSH
Garba et al. [9]	Nigeria	Cross‐sectional	83 HbSS vs. 83 HbAA	Reproductive age	HbSS	AMH

*Note*: Shandley 2024a (Br J Haematol) and Shandley 2024b (Fertil Steril abstract) represent the same cohort; data have been consolidated.

Abbreviations: AFC, antral follicle count;AMH, anti‐Müllerian hormone; BMT, bone marrow transplantation; COH, controlled ovarian hyperstimulation; DOR, diminished ovarian reserve; E2, oestradiol; FSH, follicle‐stimulating hormone; GPx, glutathione peroxidase; HbAA, normal haemoglobin genotype; HbSS, homozygous sickle cell anaemia; HU, hydroxyurea; MDA, malondialdehyde; POI, premature ovarian insufficiency; SCA, sickle cell anaemia; SCD, sickle cell disease; SOD, superoxide dismutase; VOE, vaso‐occlusive episode.

**TABLE 3:** Quantitative ovarian reserve outcomes in women with sickle cell anaemia.

**Study**	**AMH findings**	**AFC findings**	**FSH findings**	**Fertility preservation/clinical outcomes**	**Overall interpretation**
Kopeika et al. [4]	Significantly lower AMH in SCD (7.6 vs. 13.4 pmol/L; *p* < 0.001)	Not reported	Not reported	—	Evidence of diminished ovarian reserve in SCD
Shandley et al. [10]	Lower AMH in SCD (2.23 vs. 4.12 ng/mL; *p* < 0.001); 63% lower with current HU	Not reported	Not reported	—	SCD and HU associated with reduced AMH
Pecker et al. [5]	19% met criteria for DOR; lower AMH associated with HU exposure	Positive correlation between AMH and AFC	FSH elevated in DOR cases	—	Early diminished ovarian reserve observed in young SCA patients
Goldenberg et al. [7]	Median AMH lower than age norms	Not primary outcome	Not reported	13% required > 1 cycle; reduced oocyte yield in some	Suggests compromised reserve affecting fertility preservation
Adewunmi et al. [15]	Significantly lower in HbSS (1.52 vs. 3.80 ng/mL)	Significantly lower in HbSS (12 vs. 15)	Not assessed	*p* < 0.001 for AMH and AFC; negative correlation with age	Reduced ovarian reserve in HbSS; age‐related decline
Elchuri et al. [8]	Undetectable AMH in all BMT; 24% HU group had DOR	Not reported	Elevated FSH in POI cases	89% BMT had POI	Treatment‐related ovarian compromise
Garba et al. [9]	Significantly lower AMH in HbSS (3.64 vs. 7.35 ng/mL; *p* < 0.001)	Not reported	Not reported	—	Reduced ovarian reserve in HbSS

*Note*: Direct comparison of AMH values between studies is limited by different assay methods and units (pmol/L vs. ng/mL; 1 ng/mL ≈ 7.14 pmol/L, though assays vary).

Abbreviations: AFC, antral follicle count; AMH, anti‐Müllerian hormone; BMT, bone marrow transplantation; DOR, diminished ovarian reserve; FSH, follicle‐stimulating hormone; HbSS, homozygous sickle cell anaemia; HU, hydroxyurea; OR, odds ratio; POI, premature ovarian insufficiency; SCA, sickle cell anaemia; SCD, sickle cell disease.

Geographical regions represented included the United Kingdom, United States, Nigeria and European multi‐centre networks. Sample sizes varied considerably, ranging from 26 participants in one study [5] to more than 150 participants in comparative ovarian reserve studies [10, 15].

Primary ovarian reserve measures included AMH, AFC and FSH. Some studies assessed ferritin as a measure of iron overload, and one study evaluated oxidative stress markers including superoxide dismutase, glutathione peroxidase and malondialdehyde [15].

Table 3 presents key quantitative results, including AMH and AFC comparisons between the SCA and control groups, the prevalence of diminished ovarian reserve (DOR), and treatment‐related outcomes.

### 3.2 Main Findings on Ovarian Reserve Markers in SCA Compared With Controls

Across the literature, women with SCA (particularly HbSS genotype) demonstrated consistently lower ovarian reserve markers than age‐matched HbAA controls.

Kopeika et al. [4] found a substantial reduction in mean AMH levels in women with SCD compared to controls (7.6 vs. 13.4 pmol/L; *p* < 0.001), with odds of low AMH 2.6 times higher in patients with SCD. Garba et al. [9] observed mean AMH values of 3.64 ± 0.65 ng/mL in women with HbSS and 7.35 ± 1.19 ng/mL in HbAA controls (*p* < 0.001).

Adewunmi et al. [15] conducted a comparative cross‐sectional study of 75 HbSS and 75 HbAA women and reported significantly lower median AMH (1.52 vs. 3.80 ng/mL, *p* < 0.001) and AFC (12.0 vs. 15.0, *p* < 0.001) in the HbSS group. Age demonstrated negative correlations with AMH and AFC among HbSS women (*r* = −0.301 and −0.360, respectively), supporting age as a major predictor of ovarian reserve in SCA [15].

Shandley et al. [10] reported lower AMH in women with SCD compared to Black comparison women (2.23 vs. 4.12 ng/mL), with differences persisting after age adjustment.

Pecker et al. [5] provided a comprehensive assessment of ovarian reserve, including AMH, FSH and AFC. A positive correlation was observed between AMH and AFC (*r* = 0.49, *p* = 0.03), confirming the utility of AMH as a biomarker in this population. Nineteen percent of participants met criteria for DOR (AMH ≤ 1.1 ng/mL with elevated FSH) [5].

Goldenberg et al. [7], in a study of fertility preservation cycles, observed median AMH levels of 2.1 ng/mL, lower than age norms. Oocyte retrieval was frequently sufficient (median 14 oocytes per cycle), although complications were common despite reduced AMH.

Table 3 summarises these findings, demonstrating consistently lower AMH and AFC in women with SCA compared to controls, with age‐related decreases in ovarian reserve markers.

### 3.3 Subgroup Analyses: Genotype and Treatment Effects

*Genotype*: The majority of comparative studies focused on HbSS genotype. Studies directly comparing HbSS with HbAA controls demonstrated significantly reduced ovarian reserve in women with HbSS [15]. Data on other genotypes (HbSC, HbSβ°) were limited and insufficient for robust genotype comparisons [9, 15].

*Hydroxyurea (HU)*: Several studies investigated treatment effects. Pecker et al. [5] found that all women with DOR were receiving HU (5/5 vs. 10/21; *p* = 0.04). Elchuri et al. [8] observed DOR in 24% of HU‐treated participants compared to none in the supportive care arm.

Shandley et al. [10] reported that current HU users had 63% lower AMH than controls, though past or never users also had reduced AMH, though less pronounced. Cause–effect relationships remain unclear, as HU exposure may reflect more severe disease [10, 16].

*Iron overload*: Shandley et al. [10] found no statistically significant difference in AMH between women with and without iron overload (ferritin ≥ 1000 ng/mL). Pecker et al. [5] similarly found no explicit correlation between ferritin and DOR.

*Oxidative stress*: Oxidative stress markers were uniquely measured by Adewunmi et al. [15]. While superoxide dismutase levels were significantly decreased in HbSS women, no significant relationship was found between oxidative stress indices and ovarian reserve markers [15]. This suggests that mechanisms beyond measured oxidative stress parameters may contribute to ovarian dysfunction in SCA.

*Bone marrow transplant (BMT)*: Elchuri et al. [8] reported undetectable AMH levels and high rates of premature ovarian insufficiency in post‐transplant patients, consistent with the established gonadotoxicity of conditioning regimens.

## 4 Summary

Women of reproductive age with SCA consistently demonstrate lower ovarian reserve indicators (AMH and AFC) compared to HbAA controls. Age appears to be a major determinant of ovarian reserve in HbSS. Treatment exposure, particularly hydroxyurea and transplant conditioning, is associated with ovarian impairment, though distinguishing treatment effects from disease severity remains challenging. Evidence on iron overload and oxidative stress is limited and inconclusive.

Despite heterogeneity in study designs and measurement approaches, the overall consistency of findings across diverse settings indicates that DOR is a potential clinical concern that warrants further longitudinal evaluation in reproductive‐age women with SCA.

## 5 Discussion

### 5.1 Summary of Main Findings

This scoping review maps the available evidence on ovarian reserve in reproductive‐age women with SCA. Across the seven included studies, the findings indicate that women with HbSS genotype demonstrate lower ovarian reserve markers compared to age‐matched HbAA controls. In particular, reductions in AMH levels and decreased AFC were consistently observed, with age demonstrating a stronger negative association with ovarian reserve indices in women with SCA compared to controls [5]. Evidence on FSH was limited and variable across studies. Subgroup analyses suggested that disease severity and treatment exposure, particularly to hydroxyurea, may be clinically relevant, though direct relationships with biochemical indicators of ovarian reserve remain incompletely defined [15, 16].

Recent cross‐sectional studies have strengthened the evidence base by demonstrating measurable differences in ovarian reserve biomarkers in women with HbSS compared to HbAA controls [9, 15]. Collectively, these findings suggest that ovarian dysfunction in SCA is likely multifactorial, potentially arising from chronic haemolysis, vaso‐occlusion, systemic inflammation, endocrine disruption or accrued disease burden rather than a single mechanistic pathway.

### 5.2 Comparison With Existing Literature

The observed reductions in AMH and AFC are consistent with the broader reproductive health literature reporting impaired gonadal function in individuals with SCA. Burubu et al. [17] showed that homozygous sickle cell patients had impaired functional ovarian reserve, supporting the biological plausibility of ovarian reserve impairment in this population. Their findings support the hypothesis that chronic hypoxia and recurrent microvascular damage may negatively affect ovarian follicular integrity [17].

While oxidative stress has been proposed as a contributing factor, recent evidence suggests that ovarian dysfunction in SCA may involve more complex mechanisms than those captured by standard oxidative stress biomarkers [15]. This aligns with research highlighting micronutrient deficiencies, including vitamin B12, in women with SCD and their potential effects on reproductive health [18].

Reproductive health in SCA extends beyond ovarian reserve to include menstrual function, fertility outcomes, hormonal regulation and broader physiological and psychosocial factors. That wider literature, which covers fragmented reproductive care and the absence of established referral pathways [19, 20], limited fertility knowledge and counselling uptake among adolescents and adults with SCD [6, 21, 22], infertility and treatment‐seeking patterns [23], fertility preservation options such as ovarian tissue cryopreservation and parental attitudes to infertility risk [24, 25], and sexual health [26], provides important clinical context but lies outside the ovarian reserve biomarker scope of this review and is therefore not examined in detail here.

Hydroxyurea exposure remains a contentious issue in reproductive outcomes. While large cohort studies such as ESCORT‐HU [27] and Ballas et al. [28] primarily measured pregnancy outcomes and safety profiles without direct AMH or AFC measurement, the possibility of gonadotoxicity, especially in adolescents and young adults, remains. Nickel et al. [29], reviewing fertility after curative therapies such as haematopoietic stem cell transplantation, demonstrated that more intensive therapies have a definitive effect on gonadal function. In comparison, outcomes with hydroxyurea appear less conclusive but warrant ongoing surveillance.

### 5.3 Implications for Clinical Practice and Research

The findings have several clinical implications. First, periodic evaluation of ovarian reserve biomarkers including AMH and AFC should be considered in reproductive‐age women with HbSS, particularly those with severe disease phenotypes or those considering disease‐modifying interventions. Although universal screening is not yet recommended given limited longitudinal data, targeted counselling is justifiable.

Second, multidisciplinary care models incorporating haematology, gynaecology, reproductive endocrinology and specialised nursing are required. According to Pecker et al. [20], fertility care in SCD requires established referral pathways rather than ad hoc arrangements. Reproductive counselling should be integrated into routine haematology follow‐up [19].

Third, early fertility preservation discussions may be appropriate, especially before curative therapies. Nickel et al. [29] emphasise the importance of planning to prevent long‐term reproductive damage. Nevertheless, ethical considerations, informed consent and cost limitations must be addressed [24, 29].

From a research perspective, longitudinal cohort studies are urgently needed to determine whether reduced AMH and AFC lead to clinically significant infertility or premature menopause. The existing evidence is predominantly cross‐sectional, limiting causal inference. Standardisation of ovarian reserve assessment protocols would enhance comparability.

Further research should investigate mechanistic pathways, including chronic inflammation, endocrine maladjustment, vascular disruption and micronutrient deficiencies. The unclear contribution of oxidative stress suggests that multiple contributing factors exist rather than a single pathogenic mechanism.

Intervention studies assessing the benefit of optimised disease control on ovarian reserve trajectories would be valuable. Given the psychosocial aspects documented in the fertility knowledge literature [6, 30], mixed‐method studies integrating biological and experiential outcomes would provide a more comprehensive understanding.

### 5.4 Limitations and Knowledge Gaps

Several limitations must be acknowledged. A key limitation is the small number of studies (*n* = 7) that assess ovarian reserve biomarkers in women with SCA, limiting the comprehensiveness of conclusions. Sample sizes were often small, and the majority of designs were cross‐sectional, preventing assessment of longitudinal decline or fertility outcomes.

Second, heterogeneity in outcome measures and laboratory assays limits comparability. AMH thresholds, AFC ultrasound protocols and reporting standards varied. Few studies comprehensively reported FSH, and cycle timing was not always documented.

Third, covariates such as BMI, parity, socioeconomic status and treatment exposure were controlled variably. Given the documented social inequities affecting people with SCD, the lack of measurement of structural determinants may affect reproductive outcomes.

Fourth, the broader literature largely focuses on fertility knowledge, pregnancy outcomes or contraceptive use rather than direct ovarian reserve measurement. While contextually valuable, this underscores a significant gap in objective biomarker development research.

Finally, most studies focused on specific geographical areas, limiting generalisability. Multi‐centre and multinational studies addressing the global burden of SCA are needed.

Overall, recent studies indicate that reproductive‐age women with SCA may have impaired ovarian reserve markers compared to controls, with age as a major determinant. Although mechanisms remain incompletely understood, the findings support proactive reproductive counselling, multidisciplinary care integration and longitudinal research to understand long‐term fertility effects.

## 6 Conclusion

This review indicates that an emergent body of evidence suggests that women of reproductive age with SCA, particularly those with the HbSS genotype, may have lower ovarian reserve indices, most consistently lower AMH and reduced AFC, than age‐matched HbAA controls, with age exerting a greater adverse effect on ovarian reserve in women with SCA. The principal limitation of this evidence is that it is predominantly cross‐sectional and drawn from small, heterogeneous samples assayed by non‐standardised methods, so neither causality nor progression to clinical infertility can be established. The priority for future research is therefore large longitudinal cohort studies that use standardised ovarian reserve assessment and complete reporting of treatment exposure, to determine whether reduced biomarkers translate into clinically significant infertility or early menopause and to inform equitable, evidence‐based reproductive counselling for women with SCA.

## Author Contributions

K.A. and S.C.A. designed the research study. A.O.F. and S.C.A. performed the research. H.O.A. and J.O.O. synthesised and analysed the data. K.A. and S.C.A. wrote the manuscript. All authors revised and approved the final version of the manuscript.

## Funding

The authors have nothing to report.

## Conflicts of Interest

The authors declare no conflicts of interest.

## Supporting information




**Supplementary Table 1**: Full Search Strategy for PubMed/MEDLINE.

## Data Availability

The data for this study are provided within the manuscript and its Supporting Information.
