# Supplementary material for: Ovarian Reserve in Women of Reproductive Age With Sickle Cell Anaemia: A Scoping Review
Source: EJHaem. 2026 Oct 1;7(5):e70410. doi: 10.1002/jha2.70410 (PMC13628153; doi:10.1002/jha2.70410)
Supplement: Supplementary file 1 [file JHA2-7-e70410-s001.docx]

Supplementary Table 1: Full Search Strategy for PubMed/MEDLINE

**Date of search:** January 31, 2026

**Search Strategy:**

("sickle cell anemia"[MeSH Terms] OR "sickle cell disease"[MeSH Terms] OR "sickle cell anaemia"[Title/Abstract] OR "sickle cell disease"[Title/Abstract] OR "HbSS"[Title/Abstract] OR "HbS"[Title/Abstract] OR "hemoglobin S"[MeSH Terms] OR "haemoglobin S"[Title/Abstract])

AND

("ovarian reserve"[MeSH Terms] OR "ovarian reserve"[Title/Abstract] OR "anti-Mullerian hormone"[MeSH Terms] OR "anti-Mullerian hormone"[Title/Abstract] OR "AMH"[Title/Abstract] OR "antral follicle count"[Title/Abstract] OR "AFC"[Title/Abstract] OR "follicle stimulating hormone"[MeSH Terms] OR "follicle stimulating hormone"[Title/Abstract] OR "FSH"[Title/Abstract] OR "fertility"[MeSH Terms] OR "fertility"[Title/Abstract] OR "fertility preservation"[MeSH Terms] OR "fertility preservation"[Title/Abstract])

AND

("women"[MeSH Terms] OR "women"[Title/Abstract] OR "female"[MeSH Terms] OR "female"[Title/Abstract] OR "reproductive age"[Title/Abstract] OR "childbearing age"[Title/Abstract])

**Limits:** English language; Humans

**Additional databases (search adapted accordingly):** Embase, Scopus, Web of Science, CINAHL
